# Abandoned frozen embryos in Argentina: a committee opinion

**DOI:** 10.5935/1518-0557.20180085

**Published:** 2019

**Authors:** Natacha Salomé Lima, Gustavo Botti, Stella Lancuba, A. Gustavo Martínez

**Affiliations:** 1 School of Psychology. Universidad de Buenos Aires. Consejo Nacional de Investigaciones Científicas y Técnicas (CONICET). Buenos Aires. Argentina; 2 Presidente de la Sociedad Argentina de Medicina Reproductiva (SAMeR); 3 Vice presidente de la Sociedad Argentina de Medicina Reproductiva (SAMeR); 4 President of the Scientific Committee of Sociedad Argentina de Medicina Reproductiva (SAMeR)

**Keywords:** frozen embryos, abandoned embryos, patients, bioethics

## Abstract

Argentina, like many other countries in the region, faces the dilemma of what to do with the increasing accumulation of frozen embryos, which are often abandoned. This report aims to address the issue of abandoned frozen embryos, following the main concerns: 1) when is an embryo considered abandoned, according to regulatory documents; 2) how can the number of cryopreserved abandoned embryos be decreased; and 3) what are the current available options for discarding these abandoned embryos. Issues concerning the fate of abandoned embryos call for a revision of the technical aspects, as well as the symbolic aspects associated with the embryos and their options for discarding. Embryo disposal is a complex and intimate decision, which depends not only, on the quality of the cryopreserved embryo, but also on the social, cultural, economic, labor and health insurance aspects. In the absence of a formal regulatory framework for such decisions in Argentina, current practices and standard procedures face significant developmental hurdles. Among future actions to be developed in the short, medium and long term by this committee are building interdisciplinary teams, fostering patient-awareness, devising guidelines, and enforcing policies regarding embryo abandonment.

## INTRODUCTION

The present study analyzes the problem of abandoned frozen embryos in Argentina from an interdisciplinary approach. Three main questions will guide the following reflection:

1. When do regulatory documents consider that the embryo has been abandoned?

2. How to decrease the number of cryopreserved abandoned embryos?

3. What to do with the embryos that are in this situation?

## TECHNICAL ASPECTS OF THE PROBLEM

The storage of cryopreserved embryos is a common practice in fertility centers. These embryos have been cryopreserved for two reasons: for being surplus from IVF -in vitro fertilization- treatments to avoid multiple pregnancy, or due to a critic decision to delay transfer waiting for decreased patient´s risk or enhanced treatment effectiveness. Some risks could be drug-induced such as hyperstimulation syndrome (OHSS), with a less receptive endometrium or high levels of plasmatic progesterone, which has a negative effect on the implantation process. Lastly, elective embryo cryopreservation might also occur in patients who undergo preimplantation genetic testing (PGT) to select euploid embryos for uterine transfer.

Regarding embryo cryopreservation, two scenarios may occur:

1- When patients cryopreserve their embryos, but do not achieve pregnancy. They may return to the fertility center for a new attempt.

2- When patients achieve pregnancy, and fulfill their family wish, and decide not to conceive more children in the future.

In the second scenario, even though a number of patients eventually decide to donate the remaining embryos, many of them may also lose contact with the facility, resulting in embryo abandonment (Lancuba *et al*., 2009). Embryo abandonment is a recurring issue, either because the family project could not be realized due to a breakup/divorce, or because the patient's indecisiveness. 

Currently, there are four facts that affect this ongoing problem:

a- Hormonal improvements used for ovarian stimulation has accomplished better genetic quality oocytes resulting in higher quality embryos. 

b- Improvements in culture media have more successfully emulated uterine tubal fluids yielding higher quality embryos.

c- Remarkable progress in vitrification technologies has improved classical slow freezing procedures, making it one of the most popular technique in the world. 

d- Most importantly, the absence of a precise legislation that regulates the fate of surplus embryos.

Consequentially, the number of cryopreserved embryos stored in Argentina´s fertility centers has increased. A survey conducted by the Argentinean Society of Reproductive Medicine (SAMeR) in April 2017 shows that in 46 out of 57 reproductive facilities (Martínez, 2017), 126 storage tanks with 54.432 cryopreserved embryos were counted. Of these, 39, 8% were cryopreserved before 2008, which means that they were stored using slow freezing procedures, a technique with low rates of success (Levi Setti *et al*., 2014; Sifer, 2014). This fact suggests that the probability of survival might be very low. 

## THE SYMBOLIC EMBRYO REPRESENTATION: MOTIVATIONS, PERCEPTIONS AND VALUES

The symbolic embryo representation, meaning the way patients perceive and refer to their frozen embryos, might change during reproductive treatments and it is determined by several conditions: the technical and medical characteristics of the patient-physician relationship; the moral, cultural and emotional aspects such as feelings, affections, fears and anxieties involved in the decision-making processes; the genetic aspects of inheritance, and normative and regulatory grounds.

These singular characterizations are related to many aspects of a patient's life: such as changes in life plans, unexpected circumstances and changes in the wish to have a child. This changing symbolism is also connected to life expectations that are impossible to predict. Furthermore, this representation may also be associated with the patient's reproductive ages; patients' reproductive choices, and the current or desired family setup.

All these components make it difficult to reach a decision regarding embryo disposal. Moreover, it is more complicated when we consider the available options: cryopreservation for future pregnancy attempts, donation or thawing and discarding are subject to changes within the fluctuations in a patient' life choices.

These factors are closely connected to the content and manner of the information patients receive during the inform consent process. From this perspective, it should be mandatory to undergo the entire process of informed consent, not as an event, but evolving with the patients throughout this dynamic transition, achieving not only a normative responsibility, but also a subjective assertion.

From the scope of the usual patient-physician relationship, it could be noticed that the decisions regarding the fate of supernumerary embryos are very dynamic and changeable over time throughout the course of fertility treatments. A patient's decision on what to do with their cryopreserved embryos is often multifactorial and highly influenced by personal values. For instance, in Argentina, the rate of abandoned frozen embryos has increased as a consequence of the greater access to reproductive treatments by a larger number of people, and it is also associated with the absence of precise legislation that regulates the disposal of in vitro embryos. Great concern has been arising in the scientific community and requires a prompt solution.

## STRATEGIES TO PREVENT ABANDONMENT OF FROZEN EMBRYOS 

Embryo disposal is a complex and intimate decision, which depends on the quality of the cryopreserved embryo, but also on the social, cultural, economic, labor and health insurance conditions. The embryo discarding decision is influenced by the changes in the life plan and family projects. Empirical research (Bruno *et al*., 2016) shows that nearly 70% of the patients delay the decision in 5 years or more. Therefore, it can be assumed that after 5 years without contact with the couples or individuals, the embryo is considered abandoned.

The Ethics Committee of the American Society for Reproductive Medicine (ASRM, 2013) states that "in no case should embryos deemed abandoned, be donated to other couples or be used in research" (p. 1849). Additionally, other researchers argue that the main problem of abandoned frozen embryos is the difficulty in making the discarding decision (Bruno *et al*., 2016). Empirical data shows that the larger the cryopreservation time gap, the greater the probability of embryo abandonment (Cattapan & Baylis, 2015; Lyerly *et al*., 2010). Another key observation is the absence of valid predictors to define or anticipate the moment when the patients are going to decide what to do with their supernumerary embryos.

Another important aspect to consider is the lack of information regarding the meaning of cryopreservation among the population. This phenomenon might question the social perception of scientific developments, and the responsibility that emerge from the buildup, transmission and dissemination of scientific knowledge to beneficiaries and the public. Additionally, there is a need to implement a unified informed consent explaining what abandonment means, for instance, in such a case where no contact has been made with the fertility center for 5 years or more.

Following a local study about the social perception of cryopreservation (Lancuba *et al*., 2009), it is estimated that 20% of cryopreserved embryos have been abandoned (loss of contact with individuals or couples for 5 years or more). Then, since the initiative to unify the informed consent, it has been estimated that 53% of the patients (individuals or couples at the beginning of the reproductive treatment) chose donation to another couple; 31% opted for thawing and discarding, and 14% of them would choose donation for research purposes.

Although this survey results are key to analyze discarding options, the differences between cryopreserved embryos awaiting future transfer and the ones that have been abandoned should be evaluated carefully. The case of abandoned frozen embryos is more complex, because there are not always written instructions for disposal, but also in cases of death, divorce, separation, failure to pay storage charges, inability to agree on future discarding, or prolonged lack of contact with the fertility center.

## SUBJECTIVE PERSPECTIVE OF CRYOPRESERVED EMBRYOS

Clinical experience shows that patients' awareness (acquaintance, education) is fundamental in reducing embryo abandonment rates. This awareness includes providing precise and accurate information; providing time for couples to reflect on written instructions before the treatment starts; selective and non-massive cryopreservation; public policies that regulate possible embryo destinations such as donation or discarding; and fostering a regulatory framework for research on embryonic stem cells.

The scope of this future research initiative should be carried out under the frame of a specific normative ground, gathering the necessary ethical requirements and protocols approved by research ethics committees, together with the establishment of authorized and qualified research teams, and informed consents signed by gamete providers. Therefore, embryonic stem cells research should be carried out under optimal guidelines and protocols would be a suitable strategy to reduce the number of abandoned frozen embryos that have been stored during the last decades in Argentinean fertility centers.

From the previous points, it is clear that there is a need to design precise and specific strategies to involve patients in decision-making processes concerning the fate of their cryopreserved embryos. In this respect, psychologists have a fundamental role in counseling patients. Psychologists, working in reproductive medicine, have three main responsibilities:

1. Provide accurate information to patients (counseling)

2. Work alongside the medical team (both sides of the patient-physician encounter)

3. Provide psycho-social care regarding fears, doubts and fantasies that might arise during the reproductive treatments.

Assisting patients involves continuous dialogue with the medical team; proving a suitable space for discussing patients' feelings, uncertainties, fears and desires about the reproductive treatment. It is also necessary for the psychologist to evaluate the effects of these general procedures over each particular case (singularity analysis).

Although regulatory arguments such as the procreation willingness, signed in the informed consent, implies a margin of choice in the frame of an autonomous decision, the psychological meaning of the embryo is shaped by moral and ethical values, social and cultural assumptions, psychological and emotional conditions which cannot be underestimated.

## MORAL AND LEGAL STATUS OF THE EMBRYO: ARGENTINE REGULATORY FRAMEWORK 

The problem of in vitro embryos, and more specifically of the ones that can be considered as abandoned, has generated many controversies also within the legal scope. The legal status of the embryo, that is, its legal nature, has its roots in the Inter-American Court of Human Rights Artavia-Murillo case (García-Sayán *et al*., 2012).

Paragraph 264 of the "Artavia Murillo *et al.* (in vitro fertilization) v. Costa Rica" case states that the protection of life, guaranteed in article 4.1 of the American Convention on Human Rights begins at conception, understood as the moment of implantation - once the embryo is implanted in the uterus. Stating that the embryo is not a person, does not suppose to treat it either as an object or as a mere thing, but it deserves a special protection, that is understood in the frames of the legislative proposal which has already been presented.

Additionally, the intervention of assisted reproductive techniques has encouraged the revision of this notion of conception, given the fact that in the past it was impossible to think of conception outside the female body. In the case of the extra-corporeal embryo, a systematic analysis of the new Argentine Civil and Commercial Code (Caramelo *et al*., 2015) and law number 26862, which regulates Assisted Reproduction (Argentina, 2013), leads to interpreting conception as pregnancy. According to the present knowledge, it would be more precise to refer to pregnancy as implantation, meaning the stage of the human embryo in which it begins to acquire rights after uterine transfer.

However, the complexities of the case promoted the development of a systemic normative analysis from the "Artavia-Murillo" case, that is part of the constitutional principles, up to the terms and conditions established by Law # 26.862 comprehensive access to medical care procedures and techniques of medically assisted reproduction (Argentina, 2013), which enables cryopreservation, gamete and embryo donation, and also cancellation of the informed consent if the patient decides to stop treatment.

It is interesting to notice that even in countries with regulations regarding the disposal of supernumerary embryos (such as in the USA), questions and controversies still occur. In Latin America (Álvarez-Díaz, 2009), there is a wide regulatory gap concerning embryo disposal, Peru and Brazil were the first countries in the region where legislation on this subject was enforced. The article 7 of the General Health Law number 26842 of Peru (Peru, 1997) states that every person has the right to resort to fertility treatments, as well as to conceive by means of assisted reproductive techniques. While the article 5 of the Brazilian Biosecurity Law (Brazil, 2005) allows scientific research with embryonic stem cells for medical purposes.

Certain ambiguities might also be found in the report developed by the Ethics Committee of the American Society for Reproductive Medicine (ASRM, 2013) regarding the discarding of abandoned embryos. This committee states that "at present, the law does not establish clear guidance on when it is lawful to discard abandoned embryos, although it is reasonable to consider that the law will treat the embryos, after a certain time as abandoned" (ASRM, 2013). Therefore, after 5 to 7 years without contact with the individual or couples it is ethically acceptable for a fertility center to consider that the embryo has been abandoned and it can be discarded.

"An individual who, or couple that, has not given written instruction for disposal, has not been in contact with the program for a substantial period of time, has not provided current contact information, and who cannot be located after reasonable attempts by the program and facility, cannot reasonably claim an ethical violation on the part of the program or facility that treats the embryos as abandoned and disposes of them." (ASRM, 2013).

Now, it is time to have a look at our initial questions: what is said about embryo abandonment in professional statements? Which are the strategies to decrease the number of abandoned frozen embryos? How to proceed in this situation?

## CLOSING REFLECTIONS AND FUTURE ACTIONS 

The problem of frozen embryo abandonment requires awareness of the social responsibility, which is needed to prevent problems and the urgent resolution of existing cases in the near future. The dilemmas that arise due to the absence of a formal regulatory framework in line with the clinical practice of assisted reproduction has gathered experts from different disciplines to reflect on strategies, future actions and standardization processes.

Medical and biological teams are currently developing strategies to selectively cryopreserve high-quality embryos in a more controlled procedure through scientific innovation.

The destiny of supernumerary embryos, such as when the patient decides to discard them, has not yet been resolved in the current regulations. The insufficient state commitment results in the absence of formal legislation. This regulatory gap imposes a greater responsibility on fertility centers and in their self-regulation. Many dilemmas related to cryopreserved embryos arise in healthcare settings; abandoned frozen embryos result in a major problem.

Among future actions to be developed in the short, medium and long term are building interdisciplinary teams; fostering patients' awareness, design of guidelines and policy enforcement regarding embryo abandonment. These actions imply the need to reflect on the concept of embryo abandonment, establishing procedures, protocols and guidelines; revise the dynamics of unified informed consents; evaluate whether the disposal options: donation to others and donation to research are the options that best represent the needs of individuals, couples and fertility centers.

Another side of the problem involves the revision of the intensity of ovarian stimulation procedures, aimed at reducing the number of supernumerary embryos in Argentinean fertility centers. Finally, it is worthwhile to think if the problem of abandoned frozen embryos does not force a paradigm shift in the strategies used so far in the country.
